# Insulin-Like *ILP2* Regulates Trehalose Metabolism to Tolerate Hypoxia/Hypercapnia in *Tribolium castaneum*

**DOI:** 10.3389/fphys.2022.857239

**Published:** 2022-04-20

**Authors:** Yuan-Yuan Wang, Xin-Yu Zhang, Xue-Rui Mu, Xian Li, Min Zhou, Yue-Hua Song, Kang-Kang Xu, Can Li

**Affiliations:** ^1^ Guizhou Provincial Key Laboratory for Rare Animal and Economic Insect of the Mountainous Region, College of Biology and Environmental Engineering, Guiyang University, Guiyang, China; ^2^ Institute of South China Karst, Guizhou Normal University, Guiyang, China

**Keywords:** *Tribolium castaneum*, insulin pathway, carbon dioxide induced hypoxia stress, *ILP2*, trehalose metabolism

## Abstract

RNAi was used to downregulate the expression of insulin-like peptides (*ILP2*), with air-modulation, and high-concentration CO_2_ stress, in the larvae of *Tribolium castaneum*. We assessed the changes in carbohydrate-related content, trehalase activity, and the expression levels of trehalose pathway genes. And pupation, adult emergence, pupation rate, and mortality were assessed. There was a significant change in the expression of *ILPs* in *T. castaneum*, at a certain concentration of CO_2_. *ILP2* RNAi did not alter the trehalose content significantly, however, the glycogen and glucose content increased significantly. High-concentration CO_2_ stress altered the trehalose content and reduced the glycogen and glucose content. The expression levels of *TPS* and *TRE2* were up-regulated by hypoxia/hypercapnia and *dsILP2* combination, with the increase of CO_2_ concentration, other trehalase genes begin to respond successively. *ILP2* knockout raised the mortality and reduced the pupation rate and eclosion rate in CO_2_. Understanding the insulin pathway responses to hypoxic stress induced by a high concentration of CO_2_ would further elucidate the mechanisms underlying trehalose metabolism in insects.

## Introduction

The red flour beetle, *Tribolium castaneum* (Herbst) is a coleopteran belonging to the family, Tenebrionidae. It is distributed in the warmer regions and is an extremely important universal pest of stored grains (Shelby, 1977; Campbell et al., 2010; Wei, 2013). It damages a variety of commodities (such as flour, beans, nuts, Chinese medicinal materials, even meat) and causes tremendous economic losses, because of its fast reproduction, long life span, complex eating habits, and strong adaptability. When the population density of pest is high, *T. castaneum* chestnut secretes benzoquinones, which imparts a pungent odor to the commodity that render it unfit for consumption (Campbell and Runnion, 2003). *T. castaneum* in stored products and grain is primarily controlled using fumigants and sprays, but long-term use has resulted in the emergence of resistant populations (Pimentel et al., 2010; Boyer et al., 2012; Opit et al., 2012; Perkin and Oppert, 2019). Furthermore, usage of insecticides on food is a concerned due to the safe of human health and environment would expose to harm. Therefore, it is essential to identify new and effective methods to control *T. castaneum*.

Hermetic storage technology, a chemical-free approach, is used to protect stored grains and pulses against insect pests (Kharel et al., 2019). Although some grain-feeding insects were control by air-tight packaging successfully, a number of storage pests show remarkable air resistance to hypoxia, including *T. castaneum* and *Callosobruchus maculatus*, can survive days of low oxygen treatment (Hoback and Stanley, 2001; Chi et al., 2011), which means hypoxia alone is not enough. Oxidative injury can be induced by too low O_2_ level in organisms which result in morbidity and mortality, whereas CO_2_ toxicity raises in a concentration-dependent manner (Cao et al., 2015a,b), Several studies have looked at less than 3% oxygen or more than 60% carbon dioxide is effective in controlling most eggs and adults of storage pests (Sauer and Shelton, 2002; Navarro, 2006; Azzam et al., 2010; Kharel et al., 2019). As a consequence, a modified atmospheres with depleted O_2_ accompanied by elevated CO_2_ maybe can control stored grain insect pests effectively, become an environmentally friendly alternative to fumigants, it can be used for gas controlled grain storage in the warehouses. (Cheng et al., 2012). This inhibits insect respiration, therefore, controls insect pests and enables green grain storage.

Trehalose is the most important carbohydrate in the insect hemolymph, accounting for 80–90% of the total insect hemolymph sugar, and is therefore, called the “blood sugar” of insects (Elbein et al., 2003; Chen et al., 2010). The trehalose concentration in the hemolymph is not regulated by a steady state in its body, but by a stress metabolite. A drastic change in the nutritional status or the external environment induces changes in the trehalose concentration in the hemolymph, for adapting to the environment and enabling growth (Zhu et al., 2018). It has been reported that trehalose protects *Drosophila* and mammalian cells from hypoxia and anoxic injury (Chen et al., 2003). Trehalose-6-phosphate synthase (TPS), which synthesize trehalose, is essential for insect growth and development, and overexpression of TPS increases trehalose levels and insect pest tolerance to anoxic (Chen et al., 2003; Chen et al., 2018). Trehalose plays a very important role in the physiological life activities and dealing with abiotic stresses of insects, one of the key regulatory hormones is insulin (Yu et al., 2008; Shukla et al., 2015; Tang et al., 2018). In insects, the insulin/insulin-like growth factor signaling (IIS) pathway regulates carbohydrate and lipid metabolism and it associate with trehalose. Insulin is responsible for lowering blood sugar and promotes synthesis of glycogen, fat, and protein. Multiple insulin-like peptides (ILPs) and a single insulin receptor (InR), in coordination with the other pathways, control the physiological activities in different tissues (He, 2015). Among of them, ILPs are a crucial controller of carbohydrate reserve depletion in insects. ILPs are involved in the regulation of hemolymph trehalose levels in various insects (Satake et al., 1997; Broughton et al., 2005; Xu et al., 2013). *ILP2* has been proven that regulate carbohydrate, protein, and lipid metabolism during starvation (Xu et al., 2013; Hun et al., 2019), so we chose to interfere with *ILP2* for research.

High carbon dioxide concentration being toxic to insects while also can effectively control most storage pests (Cao et al., 2019). It is an indisputable fact that changes in trehalose levels can help insects acclimatize to adverse environments, however, whether *ILP2* could counteract hypoxia/hypercapnia by regulating trehalose metabolism have rarely been reported. *T. castaneum* is a model insect used for genetic research, which has the most complete sequenced and annotated genome, thus providing the most advanced genetic model of coleopteran pests (Lorenzen et al., 2007). In our study, hypoxia stress induced by hypoxia/hypercapnia and combined with RNAi of *ILP2* were assessed for its effects on trehalose metabolism.

## Materials and Methods

### Insect Culture

The *T. castaneum,* continuously bred in the laboratory on whole wheat flour containing 5% yeast in an incubator at 28 ± 1°C and 65 ± 5% relative humidity under a constant 24 h dark (0L:24D). The *T. castaneum* were treated with 25% CO_2_ + 75% air, 50% CO_2_ + 50% air, 75% CO_2_ + 25% air and 95% CO_2_ + 5% air. The group treated with normal concentration of CO_2_ was the control group (CK). *T. castaneum* on the first day of eighth instar larvae were cultured under different levels of CO_2_ for 48 h and assay *ILP* genes (*ILP1*, *ILP2*, *ILP3* and *ILP4*) relative expression (Pan et al., 2020). *T. castaneum* on the first day of the eighth instar larvae was used in this study.

### Injection of dsRNA and Samples Collection

Premier 5.0 software was used to design specific dsRNA primers for *TcILP2*, following homologous alignment, and the production was cloned using T cloning, that dsRNA was synthesized using primers containing T7 promoter sequences, and the control *dsGFP* was synthesized similarly. *dsILP2* or *dsGFP* (200 ng of each) was injected into the soft part between the third and fourth abdominal segments of *T. castaneum* on the first day of eighth instar larvae. The larvae were collected 48 h after dsRNA (*dsILP2* or *dsGFP*) injection combined with different concentration CO_2_ treatment, and stored at −80°C. Fifteen insects each were used for the analysis of interferent-effect of dsRNA, gene expression, sugar content, and activity of the enzymes in the glucose metabolism pathways. All assays were performed in triplicate.

The mortality observed within 24 h of the injection was recorded as mechanical death. The mortality after 48 h of processing, the pupal rate, and the emergence rate were recorded.

### Total RNA Extraction and First Strand cDNA Synthesis

Total RNA was isolated from each sample by using the MiniBEST Universal Extraction Kit (TaKaRa, Dalian, China), following the manufacturer’s instructions. The RNA integrity was verified by 1% agarose gel electrophoresis, and the RNA concentration was measured by a NanoDrop2000 spectrophotometer (Thermo Scientific, Waltham, MA, United States). First-strand complementary DNA (cDNA) synthesis was performed using the PrimeScript® RT Reagent Kit (TaKaRa, Dalian, China) following manufacturer’s instructions.

### Quantitative Real-Time Polymerase Chain Reaction

The relative expression levels of *TcILP2* were assessed using qRT-PCR. The insects from each treatment were collected 48 h after dsRNA injection, for assessing the efficiency of the RNAi, using qPCR. Following *TcILP2* knockdown under different concentrations of CO_2_, the transcript levels of six genes pertaining to the trehalose metabolic pathway, including five trehalase genes (*TcTre1-1*, *TcTre1-2*, *TcTre1-3*, *TcTre1-4*, and *TcTre2*) and trehalose-6-phosphate synthase gene (*TcTPS*), were analyzed. The qPCR was carried out on a CFX-96 real-time detection system (Bio-Rad, Hercules, CA, United States) in a 20 µL reaction containing 1 µL (100 ng/μL) cDNA, 1 µL (10 µM) of each primer, 7 µL nuclease-free water, and 10 µL of GoTaq qPCR MasterMix (Promega). The reaction was performed under the following conditions: pre-incubation at 95°C for 2 min, followed by 40 cycles of 95°C for 30 s and annealing at 60°C for 30 s, with melting curves obtained at 65–95°C. Ribosomal protein L13a (RPL13a) was the internal control. Specific primers shown in Table 1. The experiments were performed in triplicate, with three technical replicates each. Relative gene expression was analyzed using the 2^−△△CT^ method (Livak and Schmittgen, 2001).

**TABLE 1:** Primers used in this study.

Application of Primers	Gene	Primer sequence (5′–3′)
qPCR analysis	*TcILP1*	F: CTG​GTC​TTC​ACC​GCA​CAT, R: GAG​GAC​CAG​AGT​TGG​GAT​T
	*TcILP2*	F:GCTGTCCACGGTATGCA, R: GAAGGAAGCGTCGTGGT
	*TcILP3*	F: CGGCAAATAGTGGACGA, R: CATTCATAATCCGGTGCC
	*TcILP4*	F: TTTGTACTGGGCTTGCG, R: GGA​AGA​ATA​CGC​CGA​ATA​C
	*TcTRE1-1*	F: AAC​CAA​ACA​CTC​ACT​CAT​TCC, R: AAT​CCA​ATA​AGT​GTC​CCA​GTA​G
	*TcTRE1-2*	F: GAA​GTA​TCG​GTT​GGC​TCG, R:GAGTGGGGTTGATTGTGC
	*TcTRE1-3*	F: CTT​GAA​CGC​CTT​CCT​CTG, R: CCA​TCC​TCG​TGG​TCA​TAA​A
	*TcTRE1-4*	F:CTACCTAAACCGCTCCCA, R: TGT​CCA​GCC​AGT​ACC​TCA​G
	*TRE2*	F: TGT​TGT​GCG​TTT​GTG​CTC, R: GGA​CGG​CTT​ATT​GTT​GTT​TA
	*TPS*	F: GAT​TCG​CTA​CAT​TTA​CGG​G, R: GAA​CGG​AGA​CAC​TAT​GAG​GAC
dsRNA synthesis	ds*ILP2*	F: AGG​CAA​TTA​CAA​CAC​GCT​C, R:GTTCTTCCAGTGAACAGGGT
	ds*GFP*	F: TAA​TAC​GAC​TCA​CTA​TAG​GGC​AGT​TCT​TGT​GAA​TTA​GAT, R: TAA​TAC​GAC​TCA​CTA​TAG​GGA​ATG​TTA​CCA​TCT​TCT​TTA​A

### Trehalase Activity and Sugar Content Following RNAi Combined With High Concentration CO_2_ Treatment

Following the dsRNA injection, the insects were cultured under different levels of CO_2_, and the samples were collected. *T. castaneum* samples were homogenized through ultrasonication, mixed using centrifugation, and suspended in PBS. The concentrations of glucose, trehalose, and glycogen were evaluated, the activities of soluble trehalase and membrane-bound trehalase were detected using absorption/emission at different wavelengths. Standard glucose curve and trehalase activity curve were prepared according to the kit instructions (Sigma-Aldrich, St. Louis, MO, United States). Trehalose was quantified using the anthrone-sulfuric acid method (Leyva et al., 2008). Glucose and glycogen content were quantified, as described previously (Zhang et al., 2017).

### Statistical Analyses

Analyses were carried out with SPSS Statistics 21 software (IBM Japan). Bars represent mean ± standard error (SE). Data were statistically evaluated using the unpaired two-tailed Student’s t test for two groups, and values were considered statistically significant when *p* < 0.05.

## Results

### The Expression of Insulin Pathway-Related Genes After High-Concentration CO_2_ Treatment for 48 h and the Different Expression of *TcILP2* Gene of *T. castaneum* After RNAi

**FIGURE 1:**
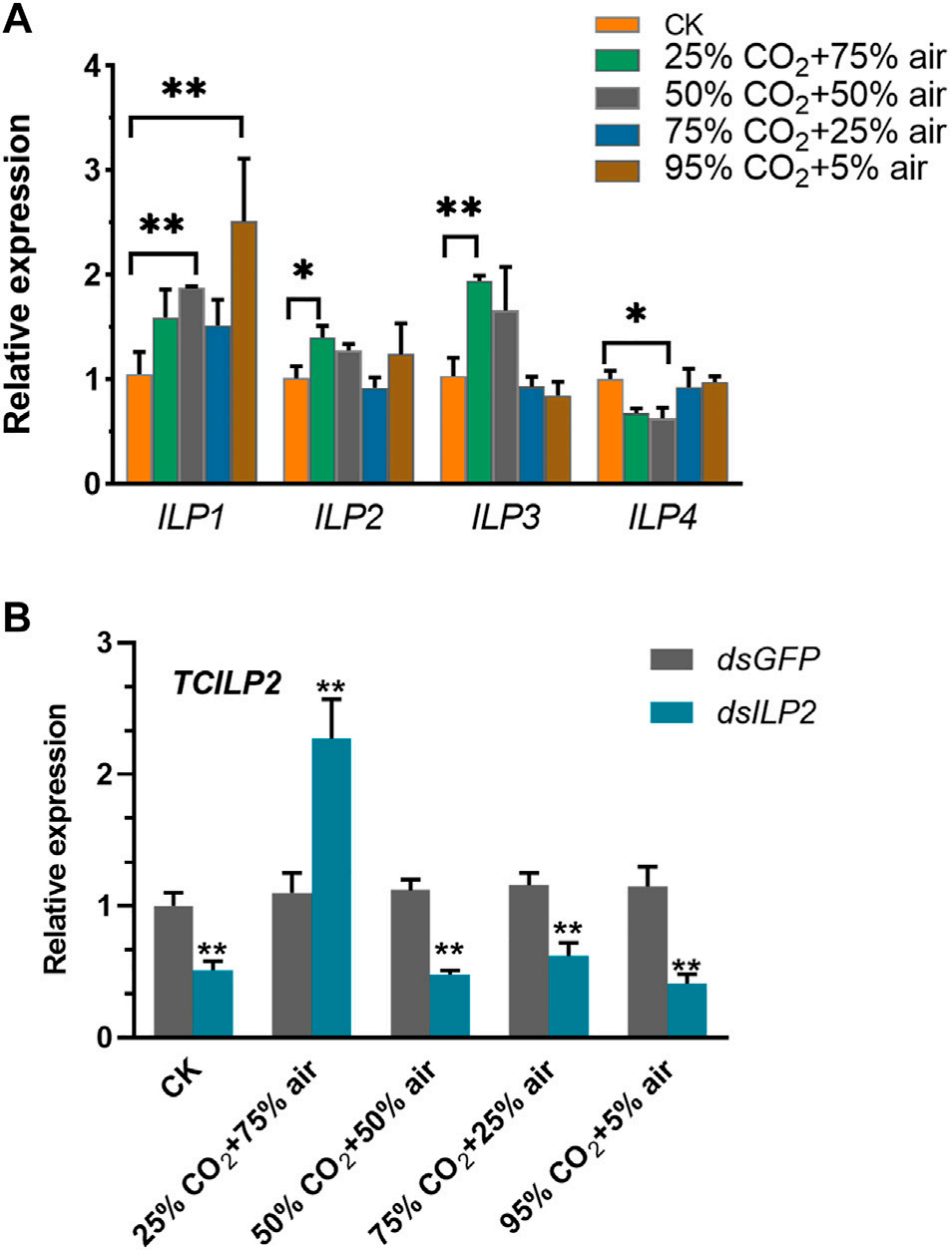
Relative expression of *TcILP* in different concentration CO_2_ treatment **(A)** and *TcILP2* **(B)** detection of RNAi interference effect after normal concentration and high concentration CO_2_ respectively. *T. castaneum* larvae were divided into three groups (each group covered 10 larvae) and used to treat with 25% CO_2_ + 75% air, 50% CO_2_ + 50% air, 75% CO_2_ + 25% air and 95% CO_2_ + 5% air, respectively. Samples were collected for 48 h treatment and mRNA expression level were detected by q-PCR. The mRNA level was normalized to that of TcRPL13a mRNA. Significant differences were identified by Student’s t-test (*p* < 0.05 was expressed by *, *p* < 0.01 was expressed by **), the same below.

High concentration of CO_2_ stress influenced the expression of the insulin pathway genes. The expression of *TcILP1* was upregulated in 25% CO_2_ + 75% air group and 95% CO_2_ + 5% air, *TcILP2* was upregulated in 25% CO_2_ + 75% air group, and *TcILP3* was upregulated in 25% CO_2_ + 75% air group, while that of *TcILP4* was downregulated in 50% CO_2_ + 50% air group (Figure 1A). The relative expression levels of *TcILP2* in response to RNAi were detected using qPCR. The expression of *TcILP2* gene was downregulated in CK group after RNAi, indicating that dsRNA successfully inhibited the expression of the target gene, *TcILP2*. When combined with high-concentration CO_2_ stress treatment, highly expressed under the 25% CO_2_ + 75% air treatment, while, *TcILP2* was significantly inhibited under other concentrations of CO_2_ (Figure 1B).

### Effects of TcILP2 Knockdown Combined With Different Concentrations of CO_2_ on the Content of Trehalose, Glucose, and Glycogen

**FIGURE 2:**
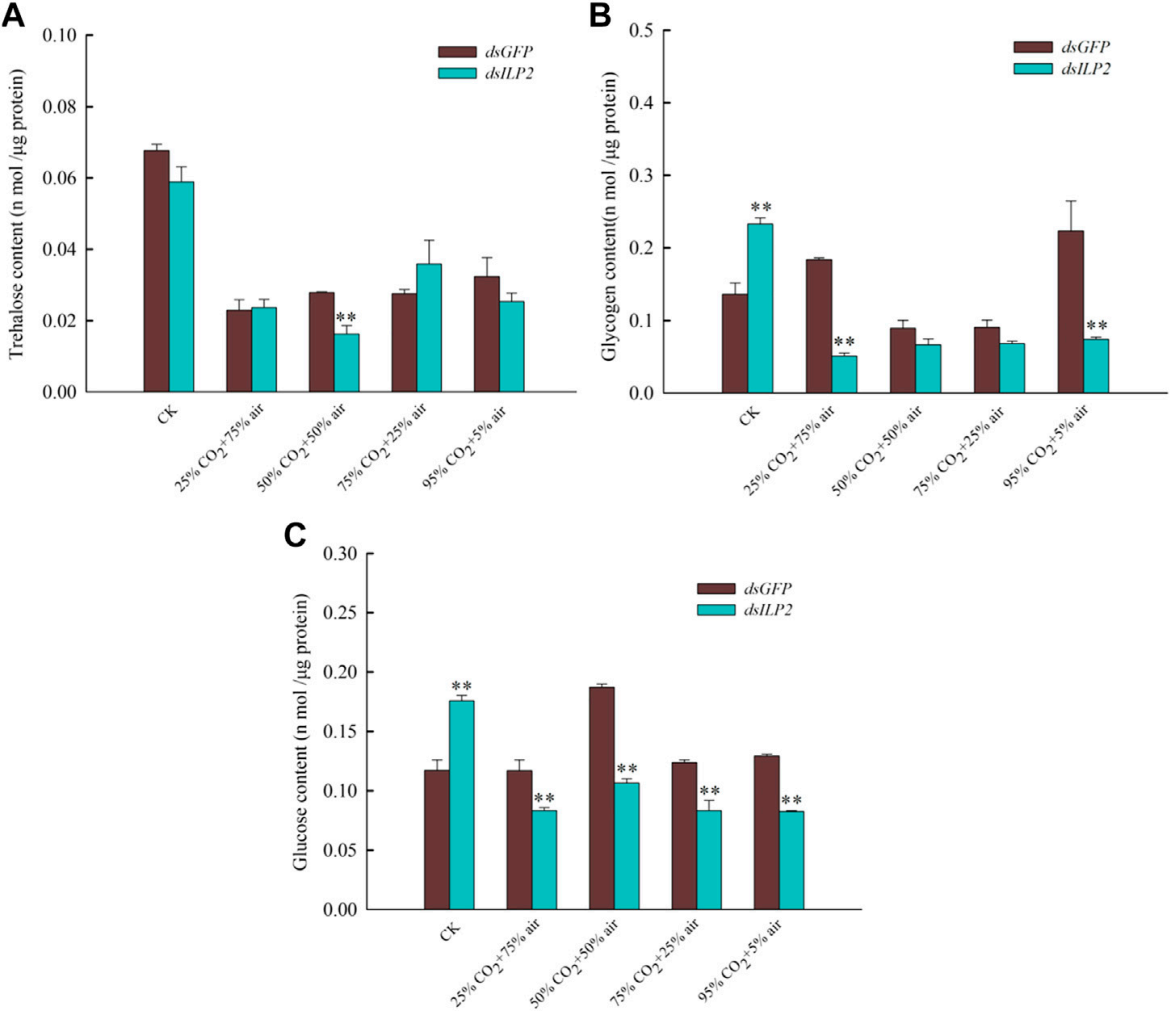
Effects of *TcILP2* knockdown on content of trehalose **(A)**, glycogen **(B)**, and glucose **(C)**. *T. castaneum* larvae were divided into three groups (each group covered 15 larvae) and injected with dsILP2 and dsGFP and combined with treatments of 25% CO_2_ + 75% air, 50% CO_2_ + 50% air, 75% CO_2_ + 25% air and 95% CO_2_ + 5% air, respectively. Insects were collected and used to detect trehalose, glycogen, and glucose content after treatments for 48 h.

Injection of *dsILP2* did not influence the trehalose content significantly (Figure 2A), but the concentrations of glycogen and glucose increased significantly (Figures 2B,C). When combined with the high-concentration CO_2_ stress treatment, the concentrations of trehalose, glycogen, and glucose decreased, however, the changes in trehalose concentration were not significant, when compared with that in the *dsGFP* group.

### Effects of TcILP2 Knockdown Combined With Different Concentrations of CO_2_ on Trehalase Activity

**FIGURE 3:**
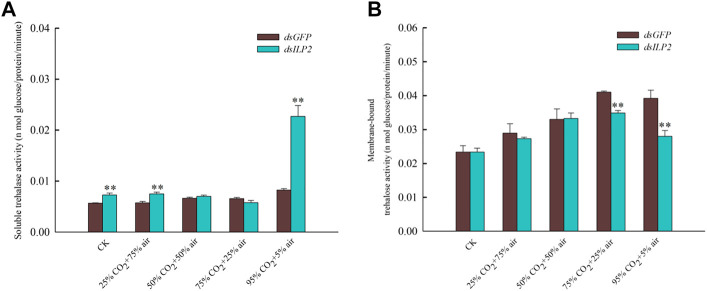
Changes of trehalase activity after RNAi combined with high concentration CO_2_. **(A)** soluble trehalase activity; **(B)** membrane-bound trehalase activity. After dsRNA (dsILP2 and dsGFP) injection, *T. castaneum* larvae were treated with 25% CO_2_ + 75% air, 50% CO_2_ + 50% air, 75% CO_2_ + 25% air and 95% CO_2_ + 5% air for 48 h. Insects were collected and divided into three groups (each group covered 15 larvae), and used to detect two trehalase enzyme activity.

The result shown that knockdown *TcILP2* could use to increase content of soluble trehalose activity (Figure 3A). When RNAi was combined with high-concentration CO_2_ treatment, the soluble trehalase activity did not change significantly under 50% CO_2_ + 50% air and 75% CO_2_ + 25% air treatments. However, it increased significantly in response to the other treatments (Figure 3A). The activity of the membrane-bound trehalase decreased significantly under the stress of 75% CO_2_ + 25% air and 95% CO_2_ + 5% air, but there was no significant change under other treatments (Figure 3B).

### Effects of *TcILP2* Knockdown Combined With Different Concentrations of CO_2_ on the Expression of Genes in the Trehalose Metabolic Pathway

**FIGURE 4:**
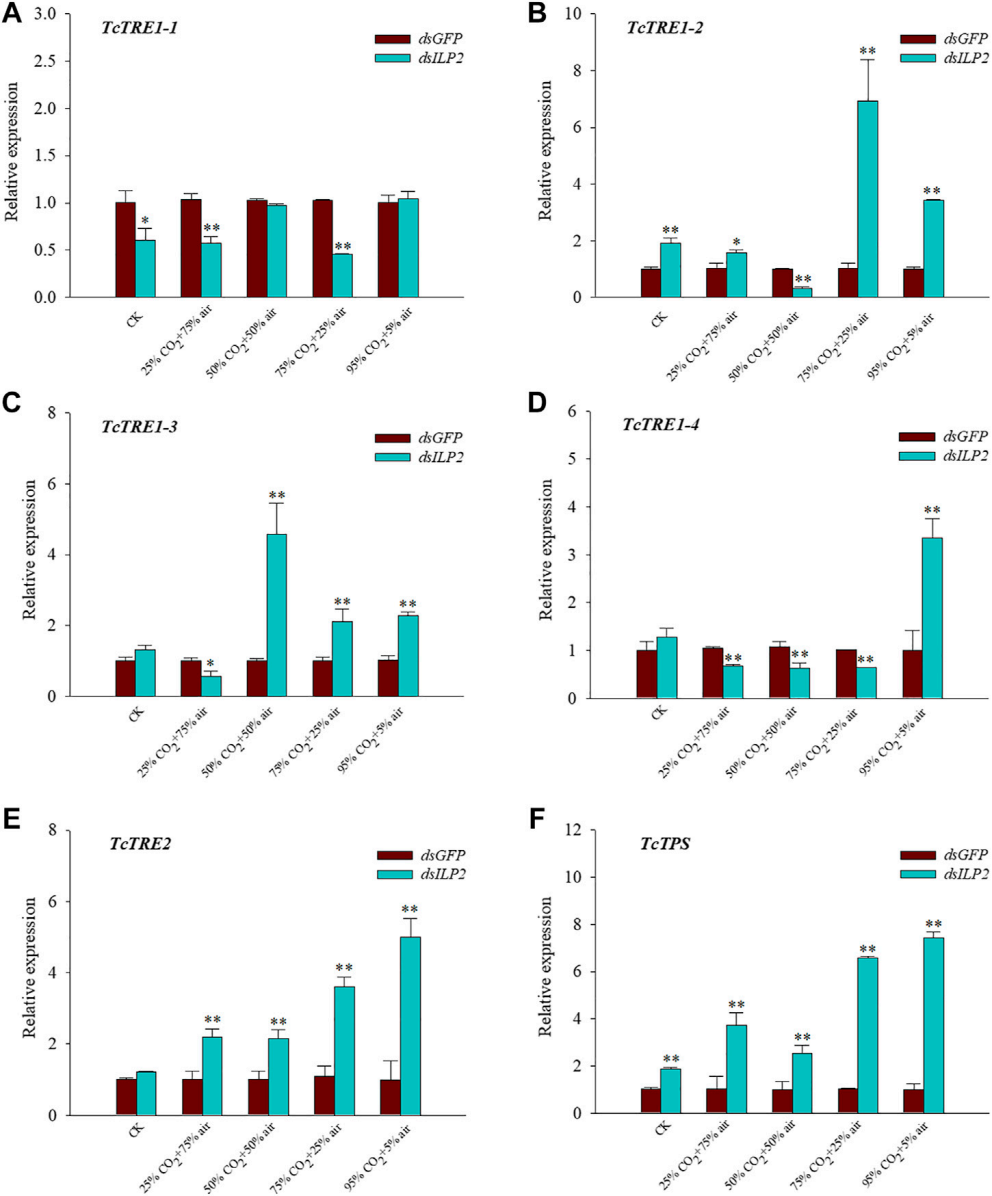
Effects of *TcILP2* knockdown on the expressions of six trehalose metabolic pathway genes. *T. castaneum* larvae were divided into three groups (each group covered 10 larvae) and injected with dsILP2 and dsGFP, and 48 h combined with treatments of 25% CO_2_ + 75% air, 50% CO_2_ + 50% air, 75% CO_2_ + 25% air and 95% CO_2_ + 5% air, respectively. Samples were collected and mRNA expression level were detected by q-PCR. The mRNA level was normalized to that of TcRPL13a mRNA. Five trehalase genes including *TcTre1-1*, *TcTre1-2*, *TcTre1-3*, *TcTre1-4*, *TcTre2* **(A–E)** and one trehalose-6-phosphate synthases gene [*TcTPS*, **(F)**].

The expression level of *TcTRE1-2* and *TcTPS* were increased significantly at 48 h after *TcILP2* inhibition (*p* < 0.05) in CK group (Figures 4B,F), on the contrary the expression level of *TcTRE1-1* was downregulated significantly (*p* < 0.05) (Figure 4A), expression level of other genes had no change. Contrary to the relative expression level of *TcTRE1-1*, *TcTRE1-3*, and *TcTRE1-4* were decreased significantly (*p* < 0.01) (Figures 4A,C,D), the relative expression level of *TcTRE1-2*, *TcTRE2*, and *TcTPS* were increased significantly (*p* < 0.01) (Figures 4B,E,F) after treatment of *dsILP2* combined with 25% CO_2_ + 75% air. The relative expression level of *TcTRE1-2* and *TcTRE1-4* were significant decrease (*p* < 0.05) (Figures 4B,D), and *TcTRE1-3*, *TcTRE2* and *TcTPS* were significant increase (*p* < 0.05) (Figures 4C,E,F) when cultivate *T. castaneum* under 50% CO_2_ + 50% air after *dsILP2* injection. 75% CO_2_ + 25% air treatments group after treatment of *dsILP2*, the expression level of *TcTRE1-1* and *TcTRE1-4* decreased significantly (*p* < 0.05) (Figures 4A,D), on the contrary, *TcTRE1-2*, *TcTRE1-3*, *TcTRE2*, and *TcTPS* were upregulated significantly (*p* < 0.05) (Figures 4B,C,E,F). As for *dsILP2* combined with 95% CO_2_ + 5% air treatments, the expression of *TcTRE1-1* only has any change, while other genes (*TcTRE1-2*, *TcTRE1-3*, *TcTRE1-4*, *TcTRE2*, *TcTPS*) increased significantly (*p* < 0.05) (Figures 4B–F).

### Effects of TcILP2 Knockdown Combined With Different Concentrations of CO_2_ for 48 h on the Death, Pupation, and Emergence of the *T. castaneum*

**FIGURE 5:**
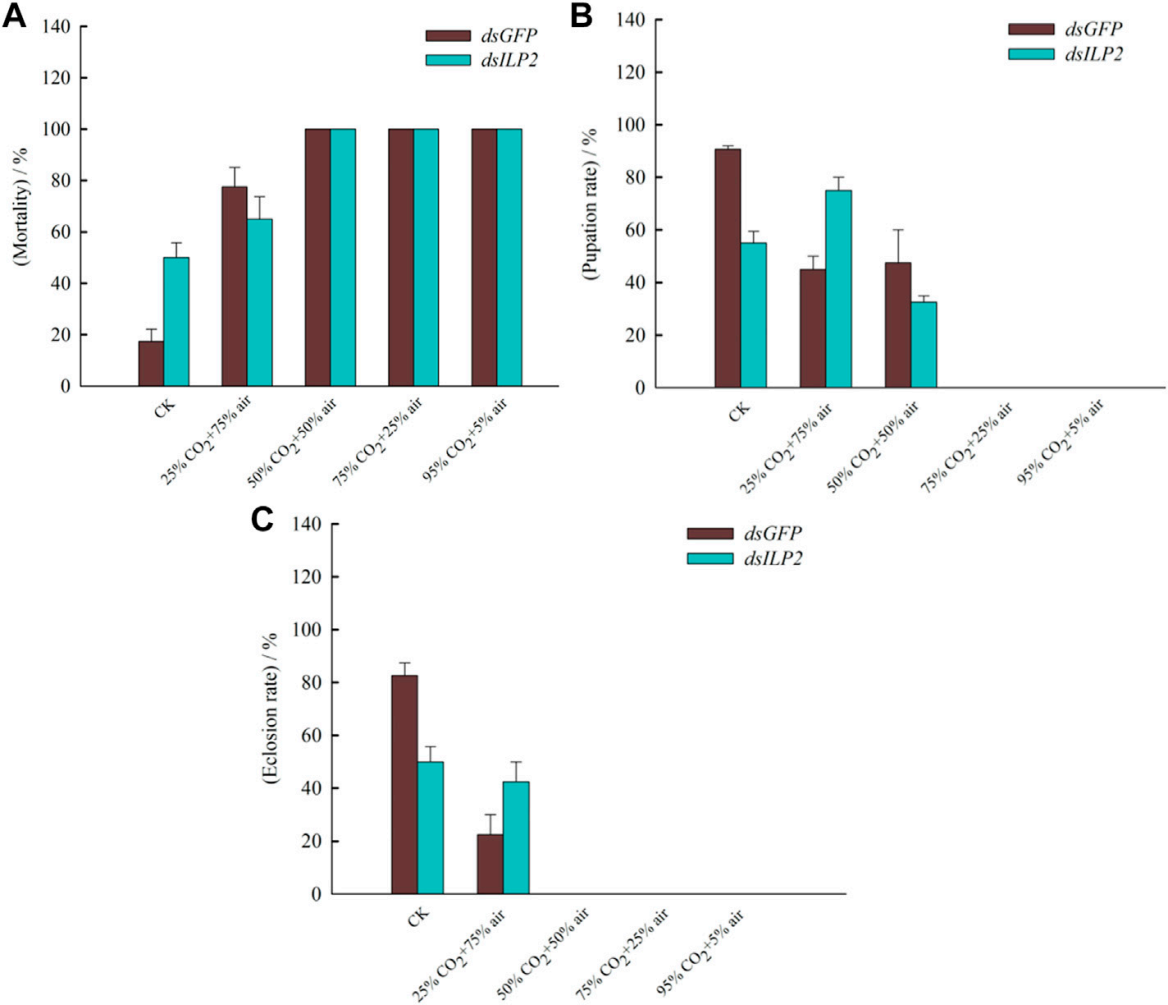
The mortality, pupation rate, and emergence rate of the red rice pirate treated with RNAi combined with high concentration CO_2_. **(A)** mortality; **(B)** pupation rate; **(C)** emergence rate. *T. castaneum* larvae were divided into three groups, each group covered 150 larvae. The samples used to be counted mortality, pupation, and emergence rate.

The mortality increased in both the *dsGFP* and the *dsILP2* treatments 48 h with the increase in the CO_2_ concentration, the mortality rate had been reached 100% at 50% CO_2_ + 50% air concentration (Figure 5A). In addition, at normal CO_2_ concentration, the mortality rate of the *dsILP2* group was significantly higher than that of the control group, however, there was no significant difference between the groups at other concentrations. The pupation rate and emergence rate decreased with the increase in CO_2_ concentration (Figures 5B,C). The pupation rate was nearly 0 at 75% CO_2_ + 20% air concentration (Figure 5B), while the feathering rate was nearly 0 at 50% CO_2_ + 50% air concentration (Figure 5C).

## Discussion

There are four ILP(*ILP1-LIP4*) genes in *T. castaneum* (Li et al., 2008), the gene expression levels of *ILP*s were changed significantly with different concentrations of carbon dioxide when *T. castaneum* treated by four air and high CO_2_ combinations. It evidenced that changes of CO_2_ concentration level have a significant effect on IIS pathway. Exposed to starvation *Drosophila*, *dilp2* was upregulated and *dilp3* was downregulated (Birse et al., 2011). *AccILP-2* might play an important role in the response to abiotic stress including light, H_2_O_2_, heavy metals (HgCl_2_ and CdCl_2_) and pesticides (dichlorvos, paraquat and cyhalothrin) in *Apis cerana cerana* (Chi et al., 2018). Those proves that insect stress resistance requires the participation of ILPs. Therefore, we inferred that the endogenous metabolites in the larvae of the *T. castaneum* under different carbon dioxide stress changed to resist adversity. In current research, *TcILP2* was inhibited significantly in CK group which indicated dsRNA effective noticeably, it is worth noting that, only 25% CO_2_ + 75% air group appear *TcILP2* upregulation significantly, it was the same with Figure 1A, it indicated at 25% CO_2_ + 75% air could still stimulate the *TcILP2* transcription when RNAi was effective. In contrast, *TcILP2* was also significantly inhibited in stress of other high CO_2_ concentrations after gene interference successfully. *AccILP-2* expression was downregulated at 44°C (Chi et al., 2018), we inferred that high stress coupled with dsRNA could also repress target gene.

RNAi of TcILP2 up-regulated glycogen and glucose levels in the hemolymph of *T. castaneum* rather trehalose levels, in line with previous reports (Nässel and Broeck, 2016; Xue et al., 2020). It suggests that *TcILP2* is responsible for downregulation glycogen and glucose levels in the *T. castaneum*. mRNA expression levels of other IIS genes elevated by the reduction of *ILP*s (Grönke et al., 2010; Walsh and Smith, 2011; Fu et al., 2016). In our results, though knockout *TcILP2* can increase *TPS* transcript level and soluble trehalase activity, trehalose content had no obvious change, thus trehalose content may be regulated by other *ILP*s in *T. castaneum*. The decrease in glycogen and glucose content levels in 25% CO_2_ + 75% air group was likely due to *TcILP2* upregulation. However, we trended towards the interaction of CO_2_ and *ILP2*, the sudden increase in CO_2_ is clearly harmful to pest insects, and explaining the dramatic rise in mortality.

The addition of hypercapnia made a complex alteration on the hypoxia response of *Callosobruchus chinensis* transcriptome, carbohydrate metabolism was one of the most highly enriched pathways for genes significantly changed (Cui et al., 2017; 2019). Furthermore, JH regulates the expression of genes encoding trehalase and *TRE* through the *ILP2* and IIS pathways, further affects trehalose homeostasis in *T. castaneum* (Ling and Raikhel, 2021). Further detected relative expression levels of trehalose metabolism genes we discovered the high expression for *TPS* and *TRE2* in any modified atmosphere. In *Drosophila*, overexpression of *TPS* was found toward increase trehalose levels and tolerance to hypoxia (Chen et al., 2003). It was reported that glucose and glycogen are used to synthesize trehalose by *TPS* in insect tissue, *TRE2* mainly resides in fat body functions to hydrolyze membrane-bound trehalase for contribute energy, and *TcILP2* is mainly in the brain and fat body (Nardelli et al., 2019; Chowański et al., 2021). Then we postulated that *T. castaneum* enhance the expressions of *TPS* and *TRE* resist hypoxia/hypercapnia under adverse conditions. Interestingly, trehalose and glucose contents were decrease rather than increase. While the effect of *TcILP2* was effectively eliminated trehalose pathway genes begin to respond successively in order to endure increasing concentrations of CO_2_ (*TRE 1-3* expressed first followed by T*RE 1-2* then *TRE 1-4*). Those results revealed, *T. castaneum* tried to strengthen the trehalose or glucose content at the transcript levels of the trehalose pathway genes, but in high CO_2_ concentrations, fat body consumes too much energy bring on the content of trehalose or glucose extremely lower than the normal.

We found that under 25% CO_2_ + 75% air mix, the *T. castaneum* can still survive partially, and the mortality rate reaches 100% at 50% CO_2_ + 50% air, 75% CO_2_ + 25% air, and 95% CO_2_ + 5% air. The matching situation was 25% under CO_2_, *dsILP2* not only has a lower mortality rate than *dsGFP*, but also a significant increase in pupation rate and emergence rate—may be attributed to the increase in *TcILP2* expression—which is similar to Xu’s research results (Xu et al., 2013). Insulin signaling pathway plays a role in the material, sugar, and energy metabolism of insects. It has important regulatory effects on various physiological processes such as metabolism, growth and development, reproduction, and stress resistance (Yuan et al., 2018; Nässel and Zandawala, 2019; Strilbytska et al., 2020; Leyria et al., 2021). Moreover, high CO_2_ stress causes hypoxia, which directly limits energy supply and free radical damage and other processes, and ultimately leads to insect death (Nystul and Roth, 2004; Helenius et al., 2009). Combination of *ILP2* knockout and hypoxia/hypercapnia could accelerate the carbohydrates consumption and affect survival and development of insects. Thus, we hypothesized that the *TcILP2* could participate in the regulation of the trehalose metabolism during hypoxia to maintain insects alive.

## Data Availability

The original contributions presented in the study are included in the article/Supplementary Material, further inquiries can be directed to the corresponding author.
